# Dynamic instability of clathrin assembly provides proofreading control for endocytosis

**DOI:** 10.1083/jcb.201804136

**Published:** 2019-08-26

**Authors:** Yan Chen, Jeffery Yong, Antonio Martínez-Sánchez, Yang Yang, Yumei Wu, Pietro De Camilli, Rubén Fernández-Busnadiego, Min Wu

**Affiliations:** 1 Centre for Bioimaging Sciences, Department of Biological Sciences, National University of Singapore, Singapore; 2 Max Planck Institute for Biochemistry, Martinsried, Germany; 3 Howard Hughes Medical Institute, Department of Cell Biology and Department of Neuroscience, Program in Cellular Neuroscience, Neurodegeneration and Repair, Yale University School of Medicine, New Haven, CT; 4 Mechanobiology Institute, National University of Singapore, Singapore; 5 Department of Neuropathology, University Medical Center, Georg-August University Göttingen, Göttingen, Germany

## Abstract

Clathrin-mediated endocytosis depends on the formation of functional clathrin-coated pits that recruit cargos and mediate the uptake of those cargos into the cell. However, it remains unclear whether the cargos in the growing clathrin-coated pits are actively monitored by the coat assembly machinery. Using a cell-free reconstitution system, we report that clathrin coat formation and cargo sorting can be uncoupled, indicating that a checkpoint is required for functional cargo incorporation. We demonstrate that the ATPase Hsc70 and a dynamic exchange of clathrin during assembly are required for this checkpoint. In the absence of Hsc70 function, clathrin assembles into pits but fails to enrich cargo. Using single-molecule imaging, we further show that uncoating takes place throughout the lifetime of the growing clathrin-coated pits. Our results suggest that the dynamic exchange of clathrin, at the cost of the reduced overall assembly rates, primarily serves as a proofreading mechanism for quality control of endocytosis.

## Abstract (precis)

Chen et al. reconstitute endocytosis in a cell-free system and show that cargo sorting requires the dynamic dissociation of clathrin during the growth phase of the clathrin-coated pit formation.

## Introduction

Functional endocytosis depends on the coordination of multiple biochemical reactions in time and space. One of these reactions is the assembly of the coat module, including clathrin and adaptor proteins (APs), which must be coordinated with the selection and sorting of cargo proteins. This coordination of cargo selection and sorting could in principle be mediated by two general mechanisms. The first of these mechanisms would be the selection of cargo-bound adaptor and coat complexes over free adaptors before their assembly into the clathrin-coated pits (CCPs). There is some evidence in favor of this mechanism. For example, it has been shown that AP2 undergoes a conformational change upon cargo binding that increases its affinity for clathrin (Edeling et al., 2006; Kelly et al., 2014). However, this mechanism would likely be error-prone due to the nonnegligible basal rate of binding between adaptors and clathrin. A second, but not mutually exclusive, mechanism would entail a process in the coat assembly machinery that monitors cargo incorporation. Such a mechanism could be achieved by a post-assembly proofreading step that discriminates between coat components that are cargo bound and those that are cargo free, followed by the removal of cargo-free coat components in a manner akin to error correction. Kinetic proofreading mechanisms have been hypothesized to exist for other biochemical reactions that require high specificity (Hopfield, 1974; McKeithan, 1995), including COPI-dependent budding from the Golgi (Goldberg, 2000). It has also been postulated to take place during endocytosis but has not been experimentally demonstrated (Bonifacino and Lippincott-Schwartz, 2003; Traub, 2009).

Clathrin assembly in living cells is heterogeneous, characterized by events with variable lifetimes that are performed through different morphological pathways (Ehrlich et al., 2004; Perrais and Merrifield, 2005; Puthenveedu and von Zastrow, 2006; Loerke et al., 2009; Scott et al., 2018). To investigate the coupling between coat assembly and cargo incorporation in a controlled, stage-specific manner, we used a cell-free reconstitution assay (Wu et al., 2010; Wu and De Camilli, 2012). We selected cell-derived plasma membrane sheets as the membrane template for assembly. These templates allow easy incorporation of exogenously transfected trans-membrane proteins for direct visualization of cargo proteins, which is challenging to implement in artificial membrane systems. By imaging the dynamics of individual clathrin triskelia, we show that a rapid exchange of clathrin takes place throughout the lifetime of the growing CCPs and that this process requires the ATPase Hsc70. In the absence of Hsc70 activity, clathrin assembles into pits but fails to enrich cargo. This requirement of clathrin exchange for the enrichment of cargo indicates that assembly does not exclusively rely on preselected coat complexes and that post-assembly proofreading is essential for productive endocytosis.

## Results and discussion

**Figure 1.**
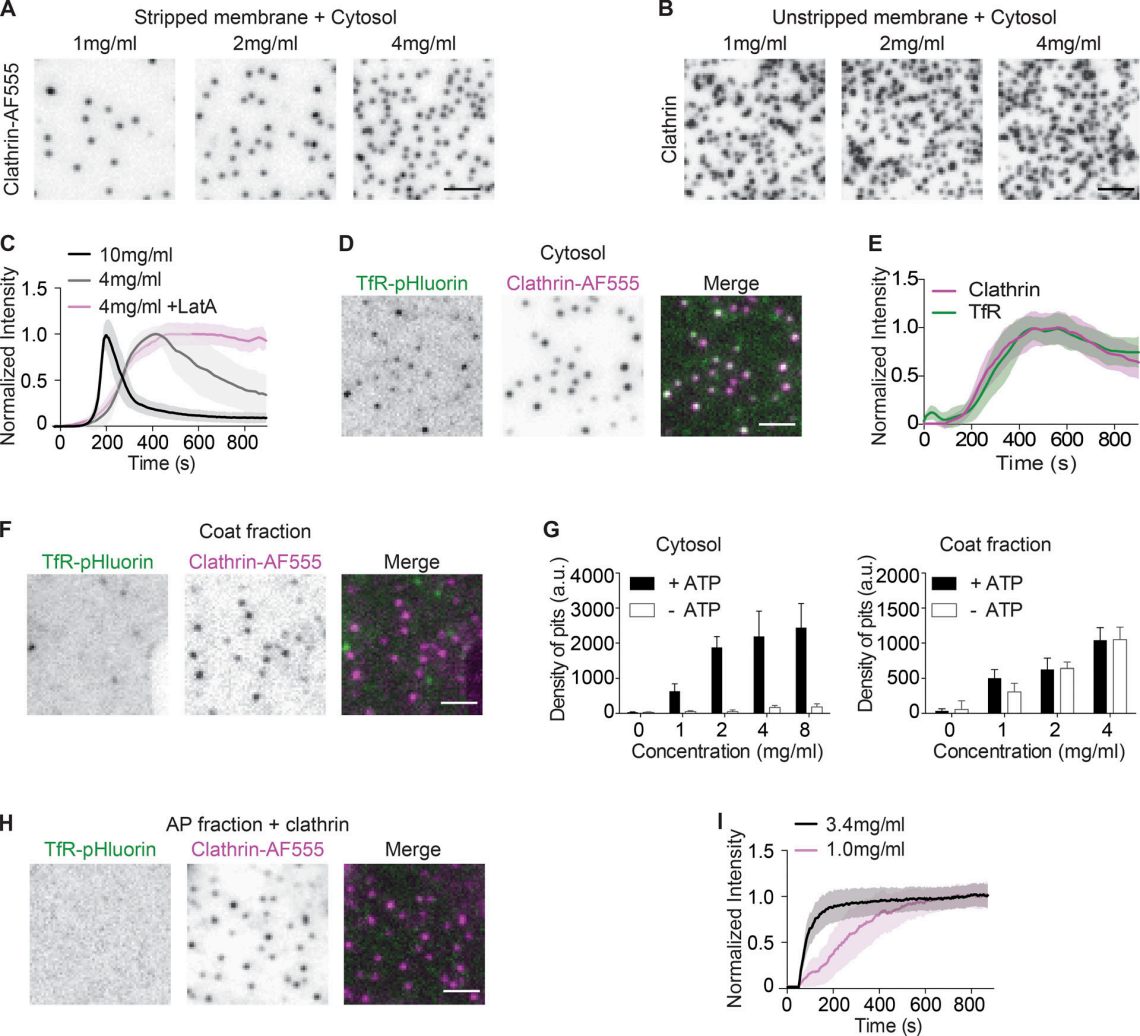
**Energetic requirement for cargo sorting, but not clathrin assembly. (A)** Representative images of CCP assembly induced by cytosol on stripped membrane sheets. CCPs were visualized by Clathrin-AF555 using TIRF microscopy. **(B)** Representative images of CCP assembly induced by cytosol on unstripped membrane sheets. CCPs were immunostained with an anti-clathrin heavy chain (X22) antibody. **(C)** Normalized intensity of Clathrin-AF555 during CCP real-time assembly induced by cytosol or by cytosol with 0.5 µM latrunculin A. **(D)** Representative images of membranes expressing TfR-pHluorin and CCPs after incubation with cytosol. **(E)** Normalized intensity of CCP real-time assembly and corresponding TfR-pHluorin puncta. **(F)** Representative images of membranes expressing TfR-pHluorin and CCPs after incubation with coat fraction. **(G)** Density of CCPs assembled with cytosol (left) or coat fraction (right) in the presence or absence of ATP. **(H)** Representative images of membranes expressing TfR-pHluorin and CCPs after AP fraction incubation. **(I)** Normalized intensity of CCP real-time assembly induced by AP fraction. Each profile of C, E, and I is the average intensity of ROI from >12 membrane sheets. Error bars indicate SD. Scale bars, 2 µm.

Membrane sheets isolated from cells are ideal for reconstitution assays because they mimic the physiological cell membrane. However, these membrane sheets also contain endogenous clathrin structures that are preserved at the time of cell lysis (Heuser, 1980; Wilson et al., 2001; Sochacki et al., 2014). These endogenous structures compromise the uniformity of reconstitution and mask de novo clathrin assembly (Moore et al., 1987; Lin et al., 1991). To overcome this limitation, we removed endogenous clathrin structures by treating membrane sheets with Tris-HCl (Keen et al., 1979), whose amine groups are predicted to break the high-affinity salt bridges between two anti-parallel clathrin heavy chains (Ybe et al., 1998). After incubating these stripped membrane sheets with brain cytosol, we observed robust, dose-dependent assembly of CCPs (Fig. 1 A), while the unstripped membranes failed to show dose dependency (Fig. 1 B).

The planar geometry of the plasma membrane sheets allows direct visualization of individual clathrin puncta at high temporal resolution using total internal reflection fluorescence (TIRF) imaging (see Video 1). The assembly of individual clathrin puncta was initiated in a relatively synchronous manner and followed similar kinetics. The assembly phase was followed by a gradual disappearance of the clathrin signals from the evanescent field (Fig. 1 C). Adding the actin polymerization inhibitor latrunculin A inhibited the decay phase, but not the assembly phase (Fig. 1 C), indicating that the decay of clathrin signals was due to the formation of actin-dependent deep invaginations. In this study, we focused on the early assembly phase, but the presence of the decay phase confirms that the maximal-intensity puncta are mature CCPs that are capable of inducing actin-dependent tubular invagination downstream of clathrin assembly (Wu et al., 2010). The rate of assembly is cytosol concentration dependent. Increased concentrations of cytosol resulted in faster overall kinetics, with a *t*_1/2_ of 73 ± 27 s for 10 mg/ml cytosol, compared with 192 ± 45 s for 4 mg/ml cytosol (Fig. 1 C).

To determine whether these de novo assembled CCPs sequestered cargo, we generated membrane sheets from cells stably expressing transferrin receptor (TfR) tagged with pHluorin. After incubating these sheets with cytosol, we observed TfR clusters that colocalized with CCPs (Video 2 and Fig. 1 D), indicating successful cargo sorting. Two-channel time-lapse imaging confirmed that clathrin assembly and TfR clustering were synchronized (Fig. 1 E). To test whether the coat assembly could be uncoupled from cargo incorporation, we then looked for conditions that would allow the reconstitution of coat assembly without cargo sorting. Interestingly, when we incubated membrane sheets with a coat fraction (Mahaffey et al., 1989; Takei et al., 1998) rather than cytosol, we observed CCP assembly on the stripped membrane, but did not observe TfR clustering (Fig. 1 F). Taken together, these observations suggest the existence of additional mechanisms that ensure simultaneous cargo sequestering during CCP assembly. Furthermore, they suggest that these mechanisms are absent during coat fraction–mediated budding.

The different requirements of these experimental conditions may shed light on the mechanisms required for cargo enrichment. One major difference we noted is that ATP is strictly required for CCP formation on stripped membranes in the presence of cytosol, but not for the coat fraction–mediated reaction (Fig. 1 G). The requirement of ATP for endocytic budding is consistent with previous in vivo and in vitro assays (Larkin et al., 1985; Tolleshaug et al., 1985; Schmid and Carter, 1990). However, the mechanisms that underlie this requirement are not clear, because the formation of CCPs on synthetic lipid membranes does not require ATP (Takei et al., 1998; Ford et al., 2001; Dannhauser and Ungewickell, 2012). We confirmed that the addition of AP fraction could stimulate the assembly of clathrin (Pearse and Robinson, 1984; Keen, 1987) on membrane sheets without the addition of ATP and in a dose-dependent manner (Fig. 1, H and I). These CCPs did not cluster TfR (Fig. 1 H). Because the CCPs assembled in the presence of coat fraction did not contain cargo, we hypothesized that an ATP-dependent reaction in the cytosol could be involved in cargo capture. In this scenario, cargo sorting, rather than coat assembly itself, would require external energy.

**Figure 2.**
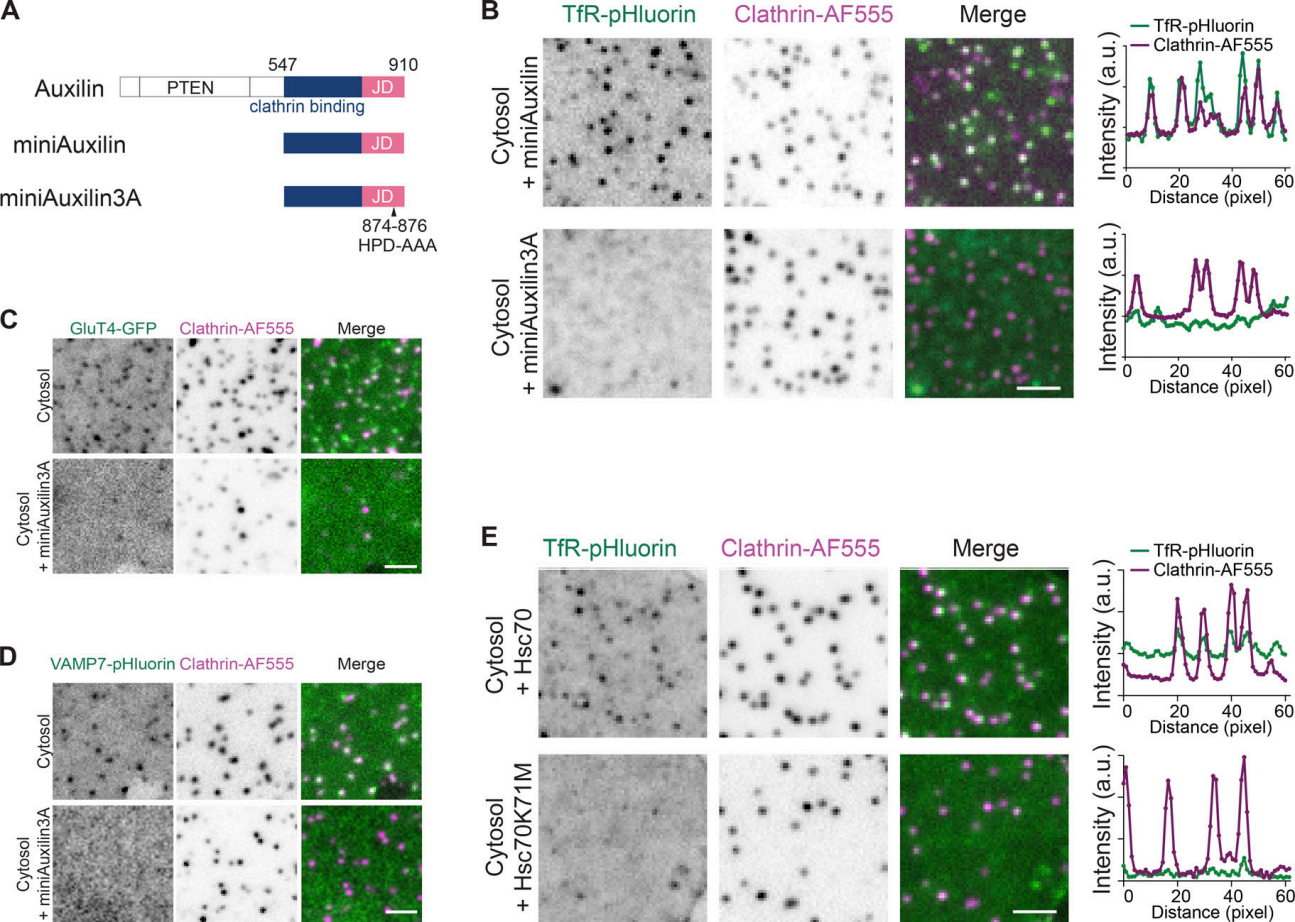
**Perturbation of Hsc70 inhibits cargo incorporation. (A)** Schematic diagram of Auxilin, miniAuxilin, and miniAuxilin3A. PTEN indicates the phosphatase and tensin homology domain and JD represents the J-domain, which contains the HPD-AAA mutations in the 3A mutant. **(B)** Representative images of membranes expressing TfR-pHluorin and CCPs after incubation with cytosol and either miniAuxilin or miniAuxilin3A. Plots represent the intensity profiles of TfR-pHluorin and CCP. **(C and D)** Representative images of membranes expressing GluT4-GFP (C) or VAMP7-pHluorin (D) and CCPs after incubation of cytosol or with miniAuxilin3A. **(E)** Representative images of membranes expressing TfR-pHluorin and CCPs after incubation of cytosol with Hsc70 or Hsc70K71M. Plots represent the intensity profiles of TfR-pHluorin and CCP. Scale bars, 2 µm.

The major ATPase in the endocytic machinery is Hsc70, which is known to uncoat clathrin (Schlossman et al., 1984; Slepnev and De Camilli, 2000). In the classic model, uncoating of clathrin mediated by Hsc70 begins after vesicle fission from the plasma membrane. This is consistent with the major recruitment of Auxilin, which in turn recruits Hsc70 upon vesicle fission (Lee et al., 2006; Massol et al., 2006). We also observed transient bursts of Auxilin2 during the assembly phase and confirmed these bursts in live cells (Fig. S1). However, it is difficult to distinguish between the functions of Hsc70 before the fission reaction from those after fission in live cells. To examine whether Hsc70 plays a role in sorting cargo during clathrin assembly before the fission reaction, we used the cofactor Auxilin and a variant that is defective in the recruitment of Hsc70 in the cell-free reaction. We produced a recombinant fragment of Auxilin that contains the clathrin-binding domain and the Hsc70-binding J-domain (miniAuxilin, Fig. 2 A; Morgan et al., 2001). Changing amino acids 874–876 in this Auxilin construct to alanines (HPD to AAA; miniAuxilin3A) markedly inhibited the interaction between Hsc70 and miniAuxilin (Morgan et al., 2001). In our cell-free reactions, the addition of miniAuxilin3A, but not miniAuxilin, to cytosolic extract interfered with TfR clustering into CCPs (Fig. 2 B). This effect was also observed with glucose transporter 4 (GluT4) and vesicle-associated membrane protein 7 (VAMP7; Fig. 2, C and D). In particular, VAMP7 has been shown to use cargo adaptors other than AP2 (Pryor et al., 2008), indicating that a lack of sorting in the absence of functional Hsc70 is not likely to be adaptor specific. To further confirm that Hsc70 was involved in cargo sorting, we used an ATPase-deficient mutant, Hsc70K71M, which has a dominant-negative effect on Hsc70 activity (Newmyer and Schmid, 2001). We observed that the addition of Hsc70K71M to the cytosol inhibited cargo sorting, whereas the addition of wild-type Hsc70 did not (Fig. 2 E), consistent with a model whereby cargo sorting requires ATP.

The uncoupling of cargo capturing and clathrin assembly, together with the critical requirement of the Hsc70/Auxilin uncoating machinery for cargo sorting, is consistent with a dynamic coat assembly model. In this model, clathrin would be continuously removed by Hsc70 during polymerization. The exchange between assembled clathrin and the bulk soluble phase requires external energy and slows down the net assembly rate. At the same time, this exchange allows the coat complex to remove assembled clathrin–adaptor complexes that are unoccupied with cargo and select for the cargo-bound complexes, thereby ensuring some quality control. Although dynamic clathrin exchange has been previously inferred from FRAP studies, these experiments typically relied on bleaching of stable clathrin structures or clusters of clathrin puncta and only very rarely examined individual growing clathrin pits (Wu et al., 2001, 2003; Hirst et al., 2008). Complicating this matter, apparent fluorescence recovery after bleaching a single growing CCP is difficult to interpret because it could be due to either growth or exchange (Ungewickell and Hinrichsen, 2007), leading to either partial or complete recovery, respectively. Indeed, the majority of FRAP studies show only partial recovery (Moskowitz et al., 2003; Hinrichsen et al., 2006; Loerke et al., 2009; Mettlen et al., 2010; Taylor et al., 2012; Kozik et al., 2013; Avinoam et al., 2015), which provides evidence for the addition of clathrin to an existing structure, but not unequivocal evidence for dissociation events.

**Figure 3.**
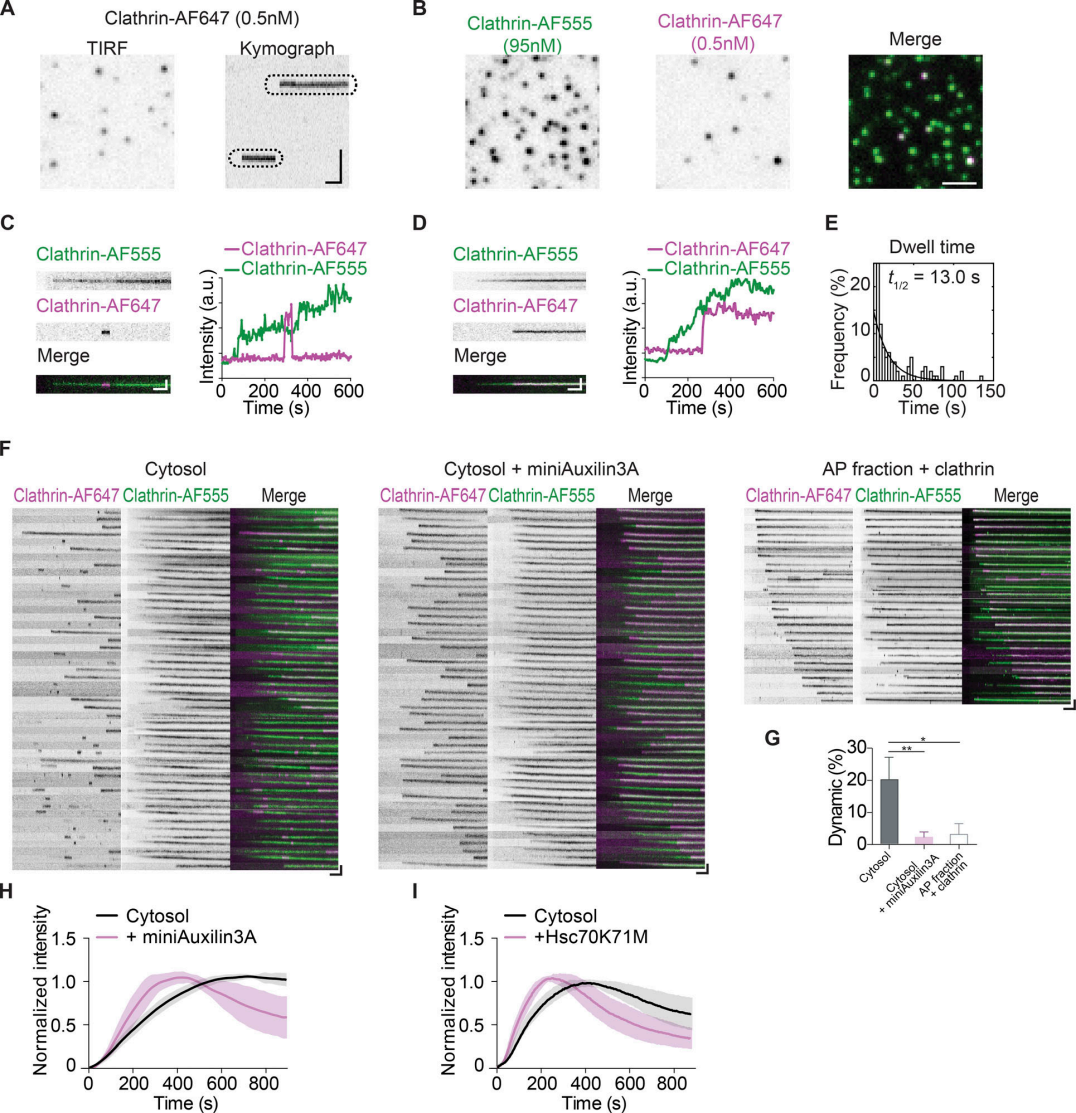
**Dynamic and stable clathrin populations on growing CCPs. (A)** A single time point TIRF image of a representative single clathrin molecule (Clathrin-AF647). The kymograph was generated from a time-lapse recording. **(B)** Single frames from time-lapse images of CCP (Clathrin-AF555), its corresponding clathrin single-molecule (Clathrin-AF647) image, and the merged image. The final concentrations of labeled clathrin are indicated. **(C and D)** Kymographs of a developing CCP with a single Clathrin-AF647 molecule, merged kymographs, and their intensity plots. Two types of single-molecule clathrin events, dynamic (C) and stable (D), are observed. **(E)** Histogram showing the distribution of the dwell times of dynamic clathrin on CCP and an exponential decay fit (R-square of fit, 0.9783; *t*_1/2_ = 13.0 s). **(F)** Kymographs of individual CCPs during cytosol (left panel), cytosol + miniAuxillin3A (middle panel), or AP fraction + clathrin assembly (right panel). **(G)** The percentage of single-molecule events during CCP development that are dynamic. Error bars indicate the SEM from three to four experiments; *, P < 0.05; **, P < 0.01, unpaired *t* test. **(H and I)** Normalized intensity of CCP real-time assembly induced by cytosol with miniAuxilin3A (H) or Hsc70K71M (I). Each profile in H and I is the average intensity of ROI from >12 membrane sheets. Scale bars, (B) 2 µm; kymograph vertical scale: (A) 2 µm, (C–F) 0.5 µm; kymograph horizontal scale, 1 min.

To test clathrin dissociation directly, we tracked the dynamics of individual clathrin molecules during assembly using very dilute (0.5 nM) Alexa Fluor 647–labeled clathrin (Clathrin-AF647) as a tracer. At this ratio, the probability that a given CCP has two Clathrin-AF647 triskelia is vanishingly low (<0.01%). Single-molecule imaging revealed two distinct clathrin populations based on their dwell time, defined as how long each molecule persisted during the experiment (Fig. 3 A and Fig. S2). One population appeared to be stable and persisted until the end of the video, while a second population of clathrin displayed a transient appearance. Because the drop in fluorescence intensity for these clathrin molecules occurred in a single frame (Fig. S2), the disappearance of signal was likely due to dissociation and not photobleaching. Indeed, due to the presence of multiple fluorophores on individual clathrin triskelia (an average of three fluorophores per triskelion), we could readily resolve a series of decreasing steps in instances of photobleaching (Fig. S3 A). Single-molecule photobleaching events were rare in our experimental conditions, but they were observed more frequently if the oxygen scavenger system was omitted (Fig. S3 B).

To determine whether the dissociation events occurred on CCPs or nonspecifically on the membranes, we included a second Alexa Fluor 555–labeled clathrin (Clathrin-AF555, 95 nM) to mark the sites of clathrin assembly (Fig. 3 B, Video 3, and Video 4). We observed that both dynamic (Fig. 3 C and Fig. S4) and stable (Fig. 3 D and Fig. S4) single-molecule events colocalized with CCPs labeled by Clathrin-AF555, indicating that the exchange events indeed took place on growing CCPs. We also observed dynamic single-molecule events away from CCPs, but these events often appeared in a single frame and they were not included in the analysis. We then quantified the dwell time of the dynamic clathrin population that was on the growing CCPs. The majority of the dynamic clathrin dissociated shortly after binding, fitting an exponential decay curve with a *t*_1/2_ of ∼13.0 s (Fig. 3 E).

We then compared the kymographs of single clathrin molecules with or without miniAuxilin3A to determine whether the effect of Hsc70 on cargo sorting depends on clathrin turnover during assembly. We observed a drastic loss of dynamic clathrin when treated with miniAuxilin3A or when assembled using an AP fraction (Fig. 3, F and G). Compared with the cytosol control, where dynamic single-molecule dissociation events were frequent (20.3% ± 3.4%, *n* = 4), the dynamic population was substantially reduced in the presence of miniAuxilin3A (2.3% ± 0.8%, *n* = 4), consistent with the role of Hsc70 in the dissociation of individual triskelia (Fig. 3 G). Similarly, we also observed minimal dissociation events (3.1% ± 1.9%, *n* = 3; Fig. 3 G) in the context of AP fraction–induced clathrin assembly, which did not enrich cargo. In addition to decreasing the proportion of dynamic single molecules, miniAuxilin3A and Hsc70K71M also increased the rate of CCP assembly (Fig. 3, H and I; *t*_1/2_: cytosol = 192 ± 45 s, Hsc70K71M = 96 ± 23 s, miniAuxilin3A = 162 ± 46 s). These results support a model whereby the dynamic exchange of clathrin is energy consuming and occurs at the cost of reduced overall assembly rates.

**Figure 4.**
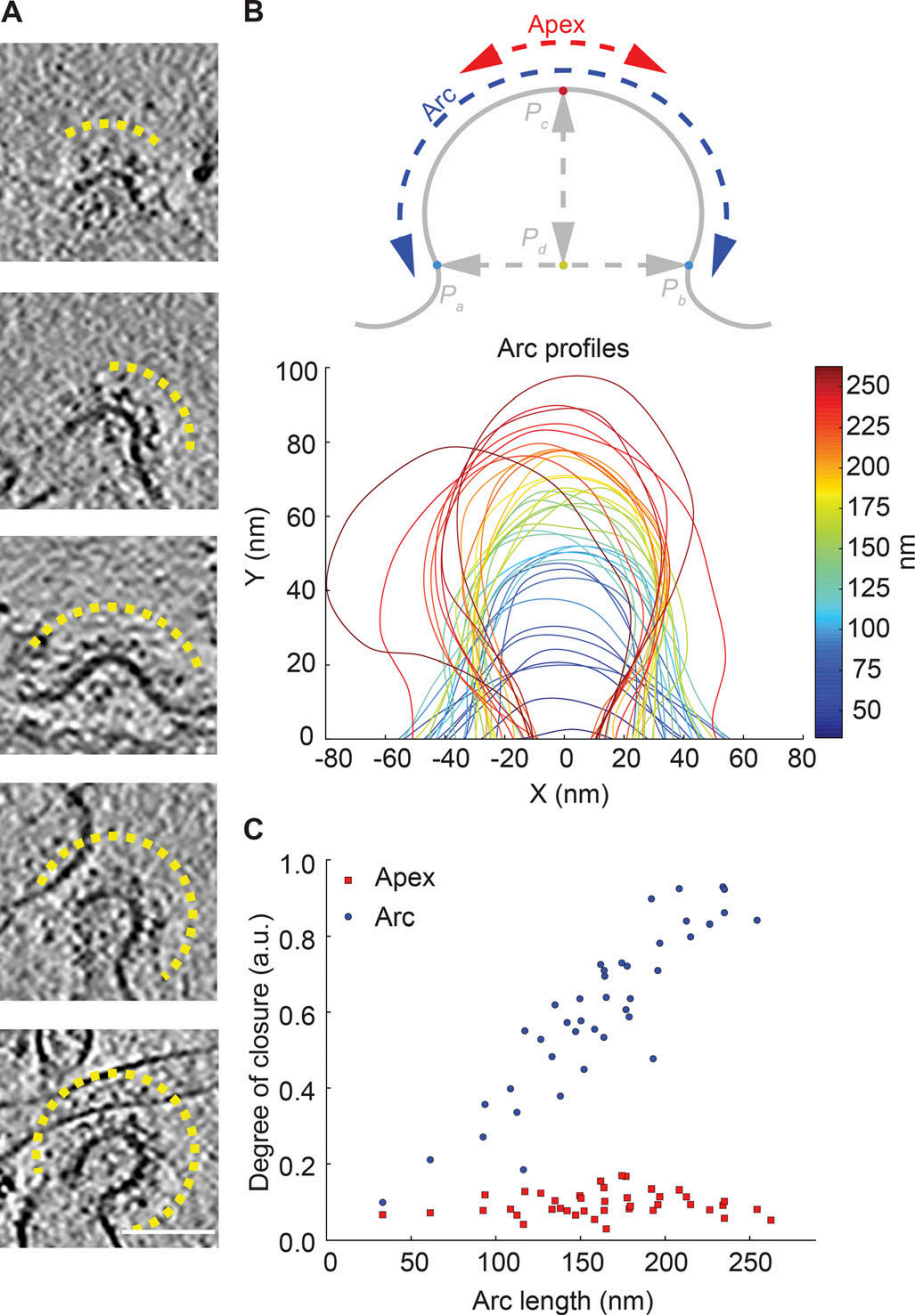
**Morphology of CCPs at different stages. (A)** Cryo-ET images of CCPs. The yellow lines highlight the CCP profiles. Scale bar, 100 nm. **(B)** Schematic representation of a CCP (top) and the individual membrane profiles of CCPs (bottom). **(C)** Degree of closure for apex and total arc for different profile lengths.

It was previously proposed that a dynamic exchange of clathrin during endocytosis is required for the rearrangement of the internal clathrin lattice structures to facilitate membrane remodeling (Heuser, 1980), and conflicting views existed for the remodeling process itself (Jin and Nossal, 1993; Wakeham et al., 2003; Kirchhausen, 2009). This idea originated from the observation of flat clathrin lattices in cells (Heuser, 1980). For flat lattices to be productive endocytic intermediates, they must at least partially dissociate. More recently, it was proposed that flat intermediates are a prerequisite for endocytosis, that membrane bending follows the completion of a flat clathrin coat assembly (Kukulski et al., 2012; Avinoam et al., 2015; Bucher et al., 2018), and that cargo could regulate this process. In this scenario, dynamic exchange of clathrin precedes and acts upstream of the acquisition of membrane curvature. To determine whether membrane bending took place during or after clathrin assembly and cargo sorting, we used cryo-electron tomography (cryo-ET) to study the membrane profiles of CCPs at different stages of assembly in our system (Fig. 4, A and B). As invagination depth and closure increased, the coated profile increased in length (Fig. S5, A and B). In contrast, neck width initially increased before decreasing again (Fig. S5 C). Importantly, local curvature at the apex did not change with increased invagination depth (Fig. 4 C). These observations suggest that the growth of the clathrin coat was synchronized with membrane invagination. Thus, exchange events take place during the membrane-budding process. We therefore conclude that dynamic exchange of clathrin is essential for cargo sorting and not an indirect result of CCP maturation through converting a flat lattice to curved CCPs.

In this study, we sought to distinguish between two potential mechanisms that ensure the coupling between coat assembly and cargo sorting: a preselected coat component model and a proofreading model. These two models can be distinguished by whether an unproductive intermediate—the assembly of coat module (including clathrin and its adaptor) without selected cargo—can be isolated. In the preselected coat component model, only cargo-bound adaptors can stimulate clathrin assembly, and therefore free clathrin–adaptor complexes would not be assembled into a growing CCP. In the proofreading model, free clathrin–adaptor complexes can be incorporated but eventually get removed. In living cells, coat assembly and cargo sorting are usually coupled; therefore, it was not yet clear whether proofreading mechanisms are needed for endocytosis. Short-lived clathrin structures can be frequently observed in vitro, and these have been interpreted as abortive endocytic events (Ehrlich et al., 2004). The presence of these presumably “abortive” events leads to the speculation that these events correspond to the elimination of intermediates that fail to load cargo. However, it was not clear whether abortive pits indeed contain less cargo. Later studies showed that a significant population of early abortive CCPs apparently assemble in a cargo-independent manner, while the late abortive CCPs disappear with increased global cargo concentrations, raising the possibility that a cargo “checkpoint” is only engaged in the late stage of endocytosis and that dynamin may serve as a candidate checkpoint protein (Loerke et al., 2009; Aguet et al., 2013). Because abortive events correspond to the simultaneous dissociation of many clathrin triskelia at the same CCP, the hypothetical checkpoints to account for their removal likely monitor the state of the CCP, which may be directly or indirectly related to cargo, or not at all. Even if they are linked to the cargo state, the synchronized dissociation of many clathrin triskelia in these abortive pits likely suggests that they monitor a threshold level of cargo, rather than directly proofreading the assembly at the level of single assembly reactions. Thus, these “CCP abortion checkpoints” based on lifetime measurements are conceptually different from kinetic proofreading reactions. Determining whether a kinetic proofreading mechanism exists during coat assembly to ensure the coupling of cargo selection and coat maturation requires direct evidence of the dissociation of individual clathrin, not evidence for abortion of entire CCPs.

**Figure 5.**
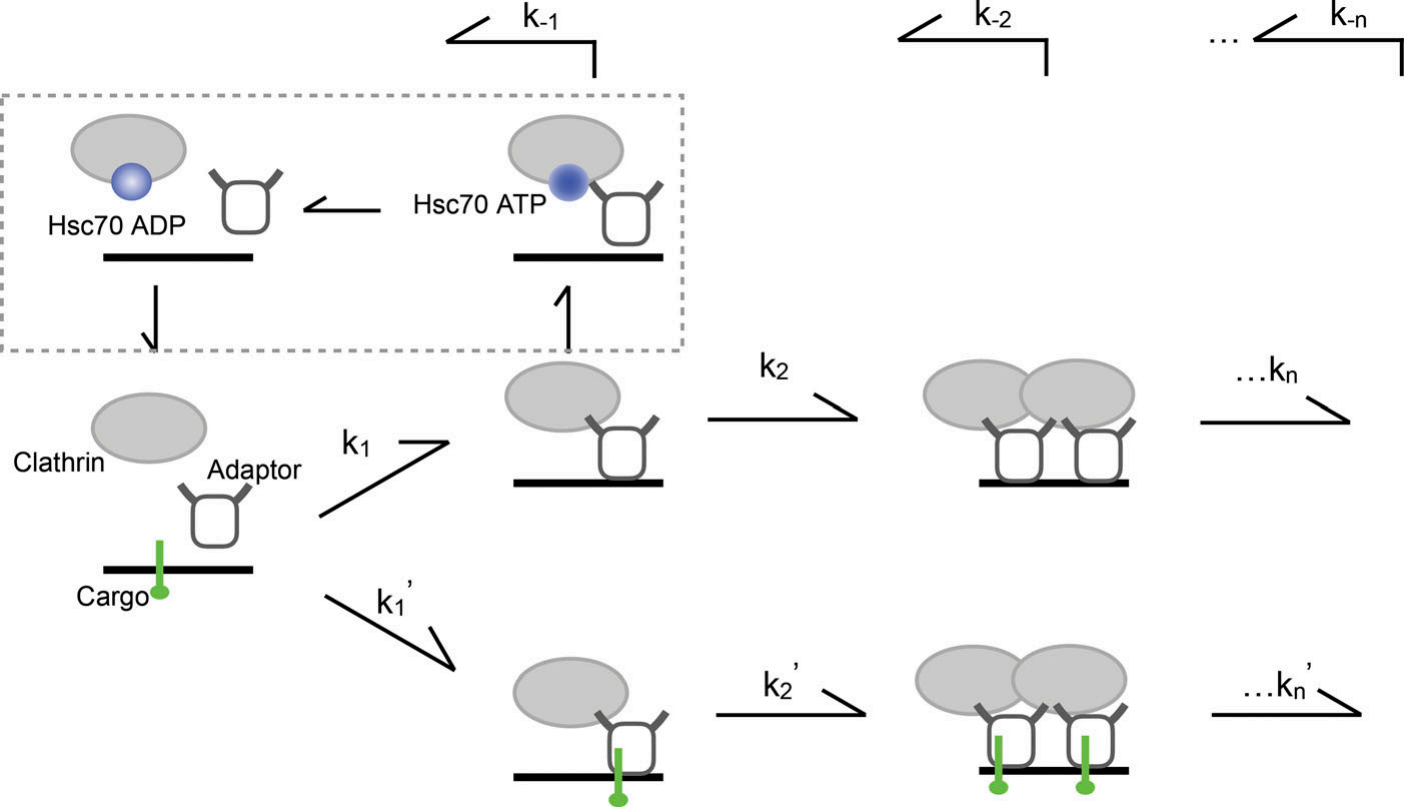
**Model of proposed role of Hsc70 in cargo sorting.** Hsc70 ATPase and its adaptor Auxilin could mediate the turnover of clathrin on the growing CCPs. In the absence of such clathrin turnover, cargo-bound and cargo-free coat components are not discriminated; coat assembly proceeds without enriching cargo. K_n_/K_n_' and K_-n_ are forward and reverse rate constants, respectively.

Our results suggest that the coordination of cargo sorting and clathrin assembly requires the dynamic exchange of clathrin during coated pit maturation (Fig. 5). The dwell time of transient clathrin is roughly 13 s, and the assembly phase lasts ∼400 s. If we assume that these pits have 60 triskelia, it means on average a single clathrin triskelion will dissociate in roughly the time it takes to add two new triskelia. We therefore infer that the dissociation events are largely localized to the edge of growing pits. This would be very similar to the dynamics of microtubule tip assembly and disassembly, but with a very different geometry. An essential component of this CCP assembly checkpoint is Hsc70 ATPase and its adaptor Auxilin, which allow dissociation events to occur. In the absence of such clathrin turnover, coat assembly proceeds without loading cargo, suggesting that cargo-bound and cargo-free coat components are no longer discriminated. The relative stability of the coat protein in the presence of the checkpoint depends on the balance between recruitment and uncoating activity. Because AP2 undergoes a conformational change upon cargo binding and increases its affinity to clathrin, cargo may stabilize the coat machinery without directly affecting the uncoating machinery. Nevertheless, we cannot exclude a direct effect of cargo binding to adaptor on the function of Auxilin/Hsc70. Such effects could result from inhibited function of Auxilin/Hsc70 (either recruitment or activity) on cargo-bound adaptor/clathrin, increased activity of Auxilin/Hsc70 on cargo-free clathrin, or both. At least in the case of AP180, a competition with Auxilin for clathrin binding has been documented (Scheele et al., 2001), which could explain how a stable adaptor might inhibit the recruitment of Auxilin. Our results highlight the importance of defining interaction affinity among multicomponent complexes in a context close to physiological conditions. Considering Auxilin/Hsc70 is recruited much more efficiently after fission than before fission, the function of Auxilin/Hsc70 must be regulated, and interactions between Auxilin/Hsc70 and coat components such as AP2 or clathrin are necessary, but not sufficient, for the action of Auxilin/Hsc70 at the growing CCPs. Therefore, simply adding Auxilin/Hsc70 to the AP fraction or coat fraction (which does contain some Auxilin; Ahle and Ungewickell, 1990) is unlikely to restore checkpoint function. Introducing mechanisms that regulate Auxilin/Hsc70 recruitment to membrane, such as those involving reversible phosphorylation of phosphoinositides (He et al., 2017), would likely be necessary to reconstitute the checkpoint.

Finally, our results also highlight the critical role of exchange rate, in addition to the nucleation and assembly steps, in dictating the lifetime of CCPs. As we have shown, changing both cytosol concentration (Fig. 1 C) and dissociation (Fig. 3, H and I) can change the rate of the net assembly. When the rate of recruitment is kept constant, such as at a given cytoplasmic composition, turnover rates could be a more physiologically relevant mode of regulation that are affected by many endocytic accessory factors, including those that are ATP dependent (such as protein kinases or lipid kinases) and ATP independent (such as protein phosphatases or lipid phosphatases). Hence, introducing an energy-dependent exchange step in an otherwise spontaneous polymerization reaction provides quality control at the cost of an overall slowing of the net assembly rate. The kinetic framework could also be relevant in understanding how a relatively small number of endocytic proteins could regulate the selection and enrichment of diverse membrane cargos (Traub, 2009).

## Materials and methods

### Reagents

Monoclonal antibody against clathrin heavy chain (X22) was purchased from Pierce (catalog # MA1-065). Porcine brains were purchased from a local butcher. Complete Protease inhibitor cocktail and GTPγS were purchased from Roche. Glutaraldehyde and paraformaldehyde were purchased from Electron Microscopy Sciences. Alexa Fluor 647–conjugated Transferrin (Tfn-AF647) was purchased from Invitrogen. All other reagents were purchased from Sigma-Aldrich, unless indicated otherwise.

GFP-Clathrin light chain was a gift from Pietro De Camilli (Yale University, New Haven, CT; originally from James Keen; Gaidarov et al., 1999; Clathrin light chain cloned in pEGFP-C1 via EcoRI and SacII sites). TfR-pHluorin was modified based on a previously published construct by Christien Merrifield (Merrifield et al., 2005). In the original construct, Super-Ecliptic pHluorin from Gero Miesenbock was used to replace GFP in Gary Banker’s jPA5-hTfnr-GFP. To confer G418 resistance, TfR-pHluorin was cut from pJPA5-TfR-pHluorin via XhoI and NotI restriction sites and inserted into the pEGFP-N1 vector (EGFP in the vector backbone was no longer present after the insertion). VAMP7-phluorin was a gift from Barbara Baird (Cornell University, Ithaca, NY; Ramezani et al., 2019; VAMP7 from Paul Roche’s EGFP-VAMP7 construct was used to replace TfR in TfR-pHluorin via EcoRI and AgeI sites). GAK-pmCherryN1 plasmid was from Christen Merrifield (Addgene; plasmid # 27695). GluT4-GFP was cloned from V5-GluT4 (Wolf Frommer; Addgene; plasmid # 18087; Yang et al., 2017). pGEX-miniAuxilin, which expresses the C-terminus of Auxilin (amino acids 547–910), and pGEX-miniAuxilin3A, which expresses the C-terminus of Auxilin (amino acids 547–910), with amino acids 874–876 mutated to alanines (HPD to AAA) mutation were kind gifts from Eileen Lafer (University of Texas Health Science Center, San Antonio, TX; Morgan et al., 2001). The Hsc70K71M mutant was produced by mutagenesis using the primer pair 5′-CTT​CGT​CCA​ATA​AGT​CGC​ATG​GCA​TCA​AAA​ACC​GTG​TTG​GTG​C-3′ and 5′-CAC​CAA​CAC​GGT​TTT​TGA​TGC​CAT​GCG​ACT​TAT​TGG​ACG​AAG-3′, with wild-type Hsc70 (gift from Lei Lu, Nanyang Technological University, Singapore) as the template. Both wild-type and Hsc70K71M were cloned into pGEX-6P1 using BamH1/Sal1 sites for recombinant protein production using primers 5′-CAA​AGG​ATC​CAT​GTC​TAA​AGG​ACC​TGC​AGT​TGG-3′ and 5′-CAA​AAG​TCG​ACT​TAG​CAG​CCA​TCA​ACC​TCT​TCA​ATG​GTG​GGC-3′.

### Cytosol preparation

Cytosol was prepared as previously described (Wu et al., 2010). Briefly, adult porcine brains were homogenized in homogenization buffer (25 mM Tris-HCl, pH 8.0, 500 mM KCl, 250 mM sucrose, 2 mM EGTA, 1 mM dichlorodiphenyltrichloroethane [DDT], and protease inhibitor cocktail). The homogenate was centrifuged at 160,000 x*g* for 2 h at 4°C. The supernatant was desalted into cytosolic buffer (25 mM Hepes, pH 7.4, 120 mM potassium glutamate, 20 mM KCl, 2.5 mM magnesium acetate, and 5 mM EGTA) using a PD-10 column (GE Healthcare Life Sciences). Protease inhibitor cocktail and 1 mM ATP were then added to the desalted cytosol. Aliquots of cytosol were flash frozen in liquid nitrogen and stored at −80°C.

### Coat fraction purification

Clathrin-coated vesicles were purified from porcine brains as previously described (Campbell et al., 1984; Takei et al., 1998; Wu et al., 2010). Porcine brains were homogenized in isolation buffer (100 mM MES, pH 6.5, 0.5 mM MgCl_2_, 1 mM EGTA, 0.02% wt/vol NaN_3_, 1 mM DTT, and 1 mM PMSF). The homogenate was centrifuged at 20,000 x*g* for 45 min at 4°C. The supernatant was collected and centrifuged at 150,000 x*g* for 1 h at 4°C. The resulting pellet was resuspended and homogenized in isolation buffer and then combined with Ficoll–sucrose solution (12.5% wt/vol ficoll-400 and 12.5% wt/vol sucrose in isolation buffer). The mixture was centrifuged at 90,600 x*g* for 25 min at 4°C. The pellet consisting of clathrin-coated vesicles was then resuspended in isolation buffer, flash frozen in liquid nitrogen, and stored at −80°C. To obtain the coat fraction, Tris-HCl, pH 7.5, was added to the clathrin-coated vesicles to a final concentration of 1 M for 2 h at room temperature to strip the coats off. The membranous vesicles were diluted with water and removed by centrifugation at 36,000 x*g* for 1 h at room temperature. The resulting supernatant (the coat fraction) was subjected to further purification steps or dialyzed into cytosolic buffer for clathrin assembly assays.

### Clathrin and AP fraction purification

Clathrin and AP fractions were purified as previously described (Campbell et al., 1984; Ahle and Ungewickell, 1986; Wu et al., 2010) from the coat fraction by gel filtration through a Tricon 10/300 Superose 6 column (GE Healthcare Life Sciences). Fractions containing clathrin or AP fraction (primarily consisting of AP2, but also containing small percentages of AP180 [Ahle and Ungewickell, 1986], AP1 [Keen, 1987], and Auxilin [Ahle and Ungewickell, 1990]) were pooled separately, dialyzed, and concentrated before being flash frozen in liquid nitrogen and stored at −80°C.

Purified clathrin was conjugated with Alexa fluorophores as described previously (Wu et al., 2010). Alexa Fluor 555 maleimide (Invitrogen A20346) and Alexa Fluor 647 maleimide (Invitrogen A20347) were used to label the purified clathrin. Conjugated clathrin were subjected to a round of assembly and disassembly to ensure functionality. The protein and dye concentrations were estimated from the absorbance at 280 nm, 555 nm, or 647 nm, respectively. The ratio of dye to protein was then calculated for each batch; values were typically in the range of 2.5–3.5 dyes per protein molecule.

### Recombinant protein production

pGEX-miniAuxilin and pGEX-miniAuxilin3A were transformed into BL21 bacteria and grown in lysongeny broth with 50 μg/ml ampicillin at 37°C overnight. The next day, the bacterial cultures were diluted 100× using lysongeny broth with ampicillin and grown to an OD600 of 0.8 before induction with 1 mM IPTG for 4 h at 25°C. The bacteria were pelleted by centrifugation at 3,000 x*g* for 30 min and then lysed by sonication in lysis buffer (PBS, 0.1% Tx-100, protease inhibitor cocktail, and 1 mM DTT). The bacterial lysates were clarified by centrifugation at 20,000 x*g* for 30 min. GST-tagged proteins were purified by incubation with 1 ml of glutathione-sepharose for 2 h at 4°C and then washed with 40 ml of lysis buffer. GST-tagged proteins were eluted using 10 mM glutathione in lysis buffer and digested with 10 U/ml thrombin (bovine plasma; Sigma-Aldrich) for 2 h at 37°C. The cleaved proteins were dialyzed into 50 mM MES-NaOH, pH 6.7, 1 mM EDTA, and 3 mM 2-mercaptoethanol overnight and further purified through a 1-ml HiTrap SP sepharose FF column as described previously (Rapoport et al., 2008). The purified proteins were concentrated, flash frozen, and stored at −80°C. Both wild-type and Hsc70K71M were grown and purified from BL21 bacteria using conditions described previously (Böcking et al., 2011).

### Membrane sheets

Membrane sheets were prepared as previously described (Wu et al., 2010; Yong et al., 2018) with minor modifications. PTK2 cells were maintained in MEM (Invitrogen) supplemented with 10% (vol/vol) FBS at 37°C in 5% CO_2_. PTK2 cells were transfected using the Neon Electroporation kit (Invitrogen), and cells stably expressing TfR-pHluorin were maintained in the same medium supplemented with 0.5 mg/ml G418 (Invitrogen). Glass-bottom dishes (MatTek) or round coverslips (No. 1.5; Marienfeld-Superior) were washed with 1 M HCl and rinsed with PBS before being coated with poly-D-lysine and washed with sterile water overnight. The indicated cells were then plated and grown for 24–48 h until confluent. Basal membrane sheets were generated by shearing the cells with several 1-s pulses of sonication in ice-cold cytosolic buffer.

### Cell-free clathrin assembly assay

For endpoint images, membrane sheets were prepared on MatTek glass-bottom dishes. Cytosol, coat fraction, or AP fraction was diluted in cytosolic buffer containing 1 mM ATP, 150 μM GTPγS, 16.7 mM creatine phosphate, and 16.7 U/ml creatine phosphokinase and added to the membrane sheets. To visualize CCPs, 10–20 nM labeled (AF555 or AF647) clathrin was added to the reaction mixture before application onto membrane sheets. For cytosol experiments without ATP, the reaction mixture was treated with 10 U of Apyrase for 30 min at 30°C before it was applied to the membrane sheets. Coat fraction was diluted into cytosolic buffer with or without 1 mM ATP, 16.7 mM creatine phosphate, and 16.7 U/ml creatine phosphokinase. 200–300 nM labeled clathrin was added for the coat fraction experiments. For assembly on membrane sheets, 1 mg/ml (∼3.5 μM) of AP fraction was mixed with an equimolar concentration of unlabeled clathrin and 200–300 nM labeled clathrin without ATP. All reactions were incubated at 37°C for 5 min and stopped by rinsing with PBS. All assembly experiments were repeated at least three times with representative sheets selected, cropped, and used to generate the figures.

For time-lapse imaging, membrane sheets were prepared on glass coverslips. The coverslips were placed into an on-stage incubation chamber (Live Cell Instrument) with a reaction chamber of 10 mm (length) by 2 mm (width) by 0.2 mm (height). Reaction mixtures were supplied into the reaction chamber by a perfusion system connected to a syringe pump (Harvard Apparatus). The perfusion pump was set to begin 10 frames after the start of imaging. The camera, laser, microscope, and pump were controlled by MetaMorph imaging software (Molecular Devices).

To track CCP assembly rate, 80–100 nM labeled clathrin (Clathrin-AF555 or Clathrin-AF647) and an oxygen scavenger system (25 mM glucose, 1.25 μM glucose oxidase, and 140 nM catalase) were added to the reaction mixture. Exposure times were 100 ms for each channel at 5-s intervals for a total of 15 min.

For imaging of single-molecule clathrin assembly, 95 nM Clathrin-AF555 was added to label all the CCP sites. Clathrin-AF647 was added to a final concentration of 0.5 nM to trace single-molecule assembly kinetics. The estimated ratio of Clathrin-AF647 to total clathrin was 1:500. The oxygen scavenging system was supplemented with 1 mM trolox and 2 mM protocatechuic acid. Exposure times were 100 ms for Clathrin-AF555 or 500 ms for Clathrin-AF647 channel at 2-s intervals for a total of 10 min.

### Fluorescence microscope

Epi-fluorescent images were acquired using a Nikon inverted Ti-S microscope equipped with a 100× oil immersion objective (CFI Plan Fluor ADH, NA 1.30; Nikon). Samples were illuminated using a light engine (Lumencor), and images were acquired using a charge-coupled device (CCD) camera (Coolsnap HQ; Photometrics). TIRF time-lapse images were acquired using a Nikon inverted Ti-S microscope equipped with a 100× oil immersion objective (CFI Apochromat TIRF, NA 1.49; Nikon) and an electron-multiplying CCD camera (Evolve 512; Photometrics) or sCOMS camera (Prime95B; Photometrics). Excitation laser (iLas2; Roper Scientific) was supplied at wavelengths of 488 nm (100 mW), 561 nm (100 mW), or 642 nm (100 mW) reflected from a multi-band dichroic mirror (Di01-R405/488/561/635; Semrock). The emitted light was filtered through multi-band emission filter (FF01-446/523/600/677; Semrock).

### Image analysis

Plots were generated using either MATLAB or Prism (GraphPad Software). Kymographs, montages, and videos were generated using ImageJ. CCPs were quantified by counting fluorescent dots in images using custom MATLAB algorithms as previously reported (Yang et al., 2017). Briefly, images of CCPs captured using an epi-fluorescent microscope were filtered using a median filter, and the local background was subtracted. Peaks that were brighter than a fixed threshold were counted as CCPs. The identified dots were overlaid with the original image for visual inspection. For each concentration and condition, the number of CCPs per image (696 × 520 pixels, 6,022 μm^2^) was counted from 30 images. CCP assembly intensity profiles were obtained from several regions of interest (ROI; 30 × 30–pixel squares) from 4 to 15 separate membrane sheets. The mean intensity for each ROI was obtained and normalized between 0 and 1 before the average normalized intensity for each time point was calculated and plotted. The SD for each time point is depicted as a shaded area on the curves. The *t*_1/2_ for each ROI was obtained by reading the time point when the normalized intensity first reached 0.5.

### Single-molecule clathrin tracking and analysis

Analysis of two-channel videos involving single-molecule data was performed as follows. CCP positions were identified from the Clathrin-AF555 channel using a custom MATLAB script as previously reported (Yang et al., 2017). The locations were then used as masks to identify Clathrin-AF647 recruited to growing CCPs. Intensity profiles were calculated as the mean intensity of 3 × 3 pixels around the identified CCP positions in the Clathrin-AF555 and Clathrin-AF647 channels. Kymographs and intensity profiles of each single molecule were generated to classify them as stable or dynamic events. All profiles were compiled and visually inspected. We defined the dwell times of dynamic clathrin single molecules as the number of frames the molecule appeared in the time-lapse videos. The dwell times of all dynamic clathrin molecules from the cytosol videos were compiled into a histogram and fitted with an exponential decay curve using the MATLAB curve fitting tool box.

### Cryo-ET

PM-GFP PTK2 cells were grown for 24 h as described above, but on poly-D-lysine–coated holey carbon films (Quantifoil). Immediately before sonication, the cells were prelysed in ice-cold water for 20 min, allowing the production of membrane sheets at a reduced sonication power to prevent damage to the carbon support. A steel disc with a central aperture was used to hold the EM grids to the bottom of the beaker during sonication. Carbon films containing membrane sheets were kept on ice for 1–2 h and then mounted on a Vitrobot plunger (FEI Company), where they were incubated with cytosol mixed with 10 nm fiducial gold particles (Aurion) for 6–8 min at 37°C and 100% relative humidity, blotted with a filter paper, and plunge frozen into liquid ethane.

Vitrified EM grids were mounted on a 626 specimen transfer holder (Gatan) cooled below −150°C and inserted into a Tecnai F20 microscope (FEI Company). The microscope was equipped with a field emission gun operated at 200 kV and a 4,000 × 4,000 CCD camera (Gatan). Tilt series were collected using SerialEM low-dose acquisition scheme (Mastronarde, 2005), typically from −60° to 60° with a 2° angular increment. The pixel size was 0.9 or 1.2 nm at the specimen level, and the defocus was set to −7 or −8 μm. The total dose was kept at <150 e-/Å_2_. Tilt series were aligned using gold beads as fiducial markers, binned three times (final voxel size of 2.7 or 3.6 nm), reconstructed by weighted back-projection with analytical weighting, and denoised by anisotropic nonlinear diffusion filtering using IMOD (Kremer et al., 1996). Tomographic images are displayed using the interpolation tool of IMOD.

### Analysis of CCP geometry by cryo-ET

CCPs were visually identified in cryo-electron tomograms by the regular pattern of the clathrin coat formed on membranes (Fig. 4 A). For every CCP, a 200 × 200–nm^2^ 2D tomographic slice containing the long axis of the invagination was chosen. Coated membranes were manually delineated with Amira 3D software to generate an initial model. Every set of segmented points was resampled 10-fold using spline interpolation. Refined profile curves were defined by three characteristic points: two border points (blue points in Fig. 4 B), *P_a_* and *P_b_*, and the point at the intermediate distance between borders, *P_c_* (red). The line defined by *P_a_* and *P_b_* and the normal to this line containing *P_c_* cross each other at point *P_d_* (green). The curves were shifted and rotated to use *P_d_* as the origin of an xy coordinate system and the line containing *P_a_* and *P_b_* as the x axis. Profiles were expressed as curves embedded in a plane: $\left[a,\;b\right]\to {\mathbb{R}}^{2},$ where $\gamma \left(a\right)=P_{a}$ and $\gamma \left(b\right)=P_{b}$ are the two border points. The following geometric parameters were analyzed for all curves to describe the geometry of CCPs at different stages of maturation without imposing a spherical approximation:

1. Arc length: length of the curve γ, $\overset{a}{\underset{b}{\int }}\left|\gamma '\left(s\right)\right|ds$ (measured in nanometers), directly related with CCP surface area.

2. Depth: maximum height of the fitted profile above the x axis (measured in nanometers).

3. Neck width: distance between the two border points (measured in nanometers).

4. Total curvature or degree of closure:$\;\overset{a}{\underset{b}{\int }}k\left(s\right)ds,$ where k(s) is the local curvature of the curve γ at point s, estimated according to Boutin (2000) (adimensional). This metric measures how closed a CCP is, with 0 representing a straight line and 2π representing a completely closed curve (fully formed vesicle).

5. Apex: a subset δ of the curve γ with *P_c_* as the central point and 20-nm length representing the local geometry at the middle part of the CCP. An apex length of 20 nm was selected because it was smaller than the total length of all CCPs analyzed, but at the same time big enough to provide robust measures.

6. Apex curvature: total curvature applied only to curve δ to evaluate the local geometry of the apex.

### Online supplemental material

Fig. S1 reports Auxilin2 recruitment during CCP assembly in live HeLa cells. Fig. S2 shows traces of both dynamic and stable single-molecule profiles of clathrin. Fig. S3 shows traces of single-molecule profiles of clathrin under stepwise photobleaching. Fig. S4 shows traces of single-molecule profiles of clathrin together with bulk clathrin. Fig. S5 shows additional cryo-ET analyses. The videos show the dynamics of clathrin (Video 1), TfR (Video 2), a single molecule of clathrin (Video 3), and a two-color merged view of bulk clathrin and single molecule of clathrin (Video 4) during CCP assembly in the cell-free reaction.

## Supplementary Material

Supplemental Material (PDF)

Video 1

Video 2

Video 3

Video 4
