# Supplementary material for: Dynamic instability of clathrin assembly provides proofreading control for endocytosis
Source: J Cell Biol. 2019 Aug 26;218(10):3200–11. doi: 10.1083/jcb.201804136 (PMC6781453; doi:10.1083/jcb.201804136)
Supplement: Supplemental Material (PDF) [file JCB_201804136_sm.pdf]

## Supplemental material

Chen et al., https://doi.org/10.1083/jcb.201804136

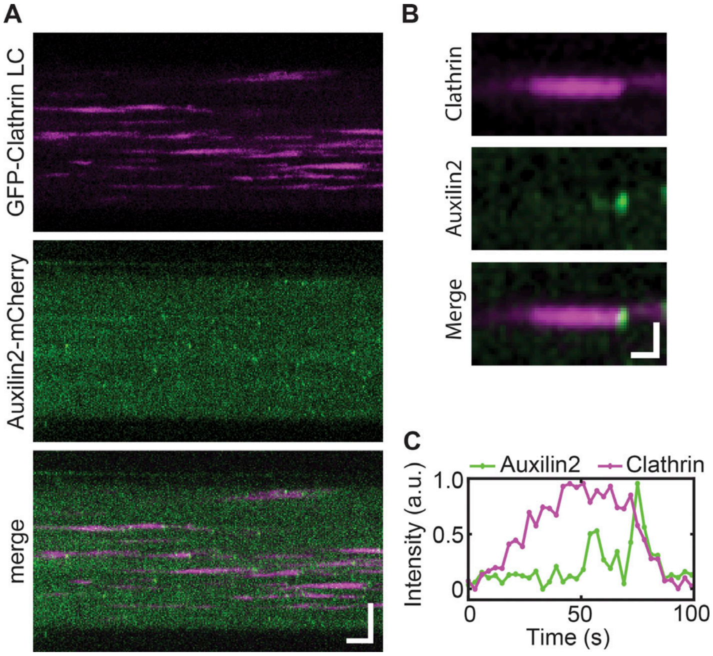


Figure S1. **Auxilin2 recruitment during CCP assembly in HeLa cells. (A)** Kymograph generated from a HeLa cell stably expressing GFP-Clathrin light chain and Auxilin2-mCherry. Images were captured under TIRF illumination for both channels. **(B)** Kymograph of a single CCP forming and pinching off with the corresponding Auxilin2-mCherry kymograph. **(C)** Normalized intensity profile of clathrin and Auxilin2 of the single CCP from B. Scale bars, kymograph vertical, (A) 5 µm, (B) 1 µm; kymograph horizontal, (A) 60 s, (B) 10 s.

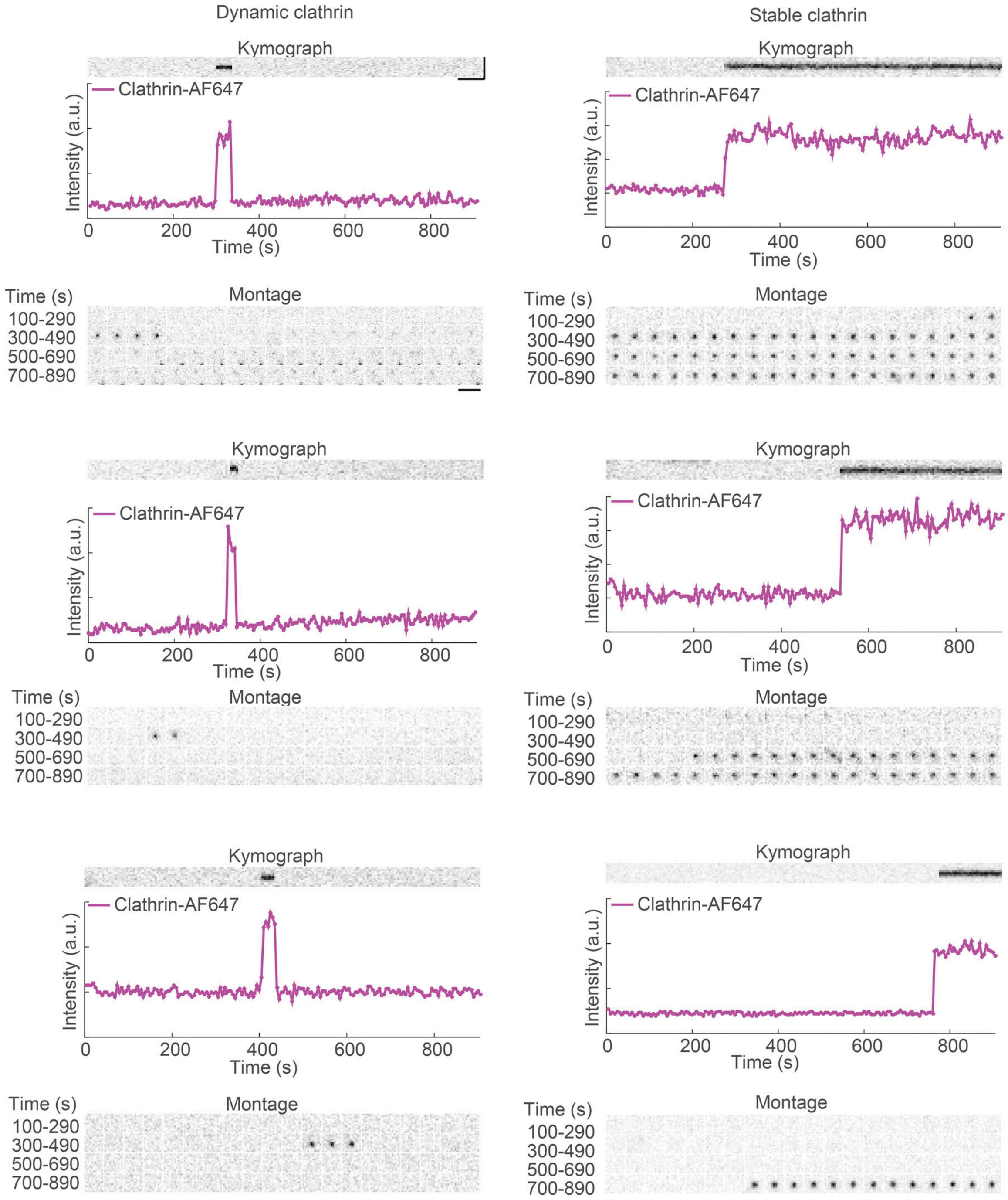


Figure S2. **Representative profiles of dynamic and stable clathrin single molecules.** Profiles for dynamic and stable single-molecule Clathrin-AF647. Kymograph (top), intensity profile (middle), and montage (bottom) of single molecules of Clathrin-AF647. Scale bars, 1 min (kymograph, horizontal); 2 µm (kymograph, vertical); and 2 µm (montage).

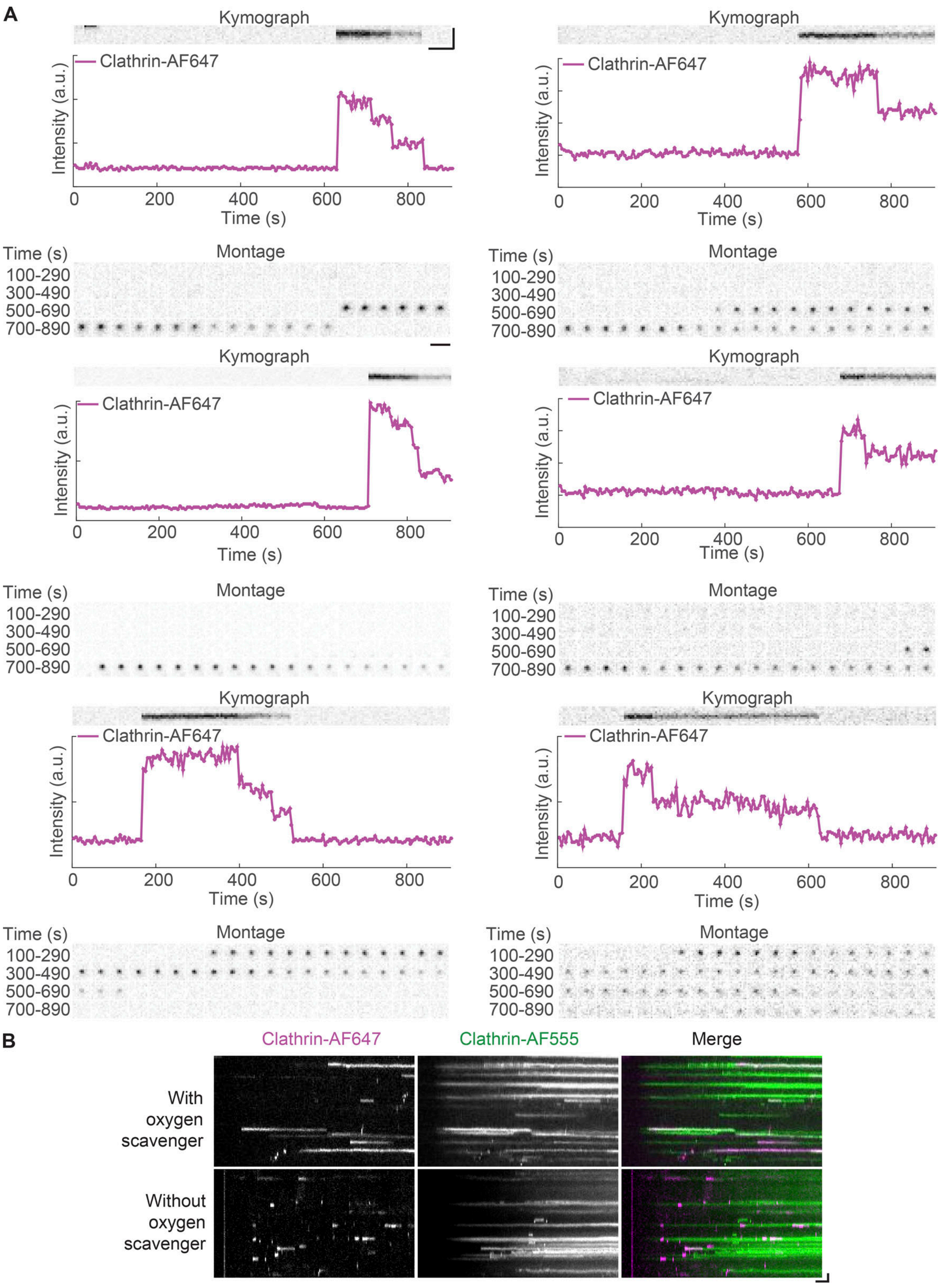


Figure S3. **Representative profiles of photobleached clathrin single molecules. (A)** Profiles of single molecules of Clathrin-AF647 undergoing possible photobleaching. **(B)** Kymographs of single molecules of Clathrin-AF647 and corresponding CCP (Clathrin-AF555). The oxygen scavenger system was included (top panel, and all other experiments unless otherwise noted) or omitted (bottom panel) in the reaction mix during live imaging. Scale bars, 1 min (kymograph, horizontal); 2 µm (kymograph, vertical); and 2 µm (montage).

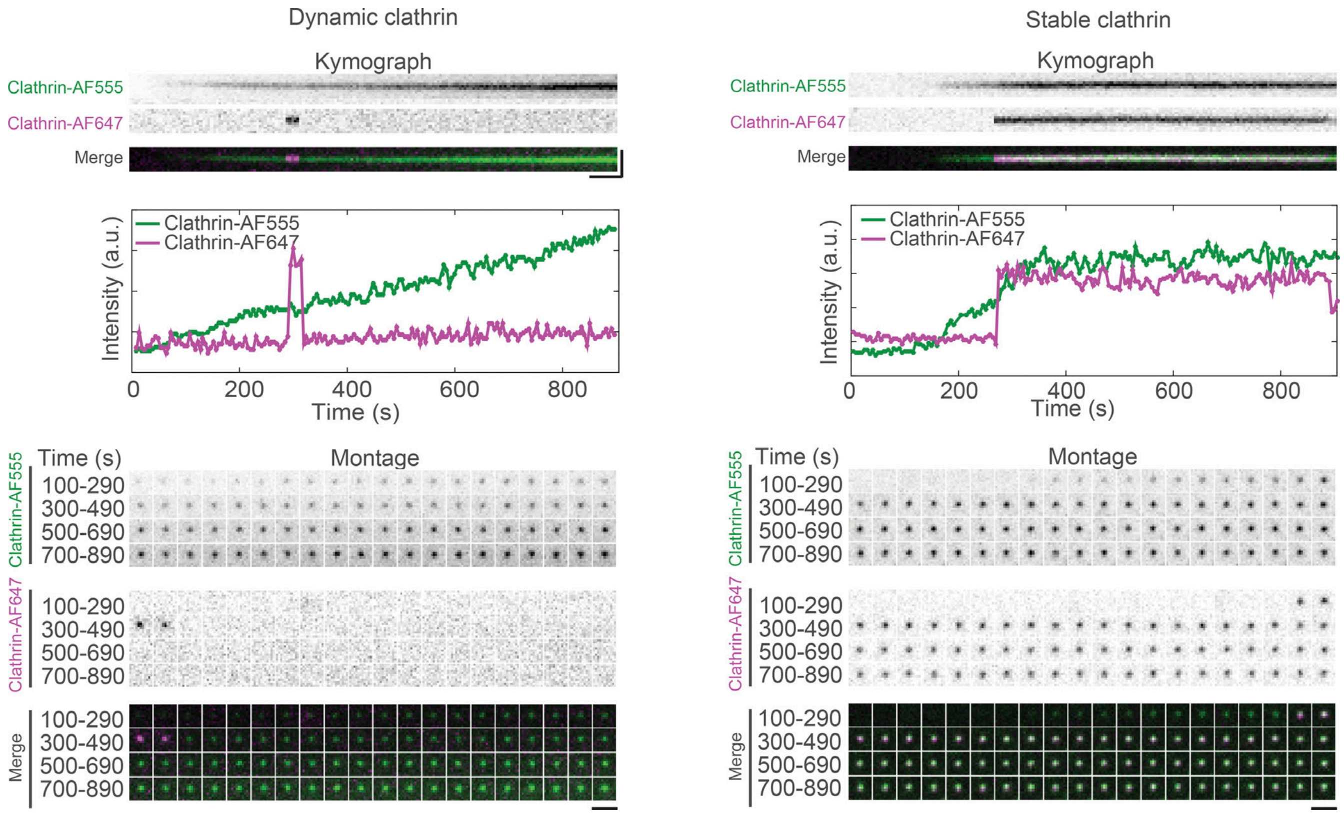


Figure S4. **Representative profiles of clathrin single molecules and bulk clathrin.** Profiles for single molecules of Clathrin-AF647 with its corresponding CCP (Clathrin-AF555). Kymograph (top), intensity profile (middle), and montage (bottom) of Clathrin-AF555 and single molecules of Clathrin-AF647. Scale bars, 1 min (kymograph, horizontal); 2 µm (kymograph, vertical); and 2 µm (montage).

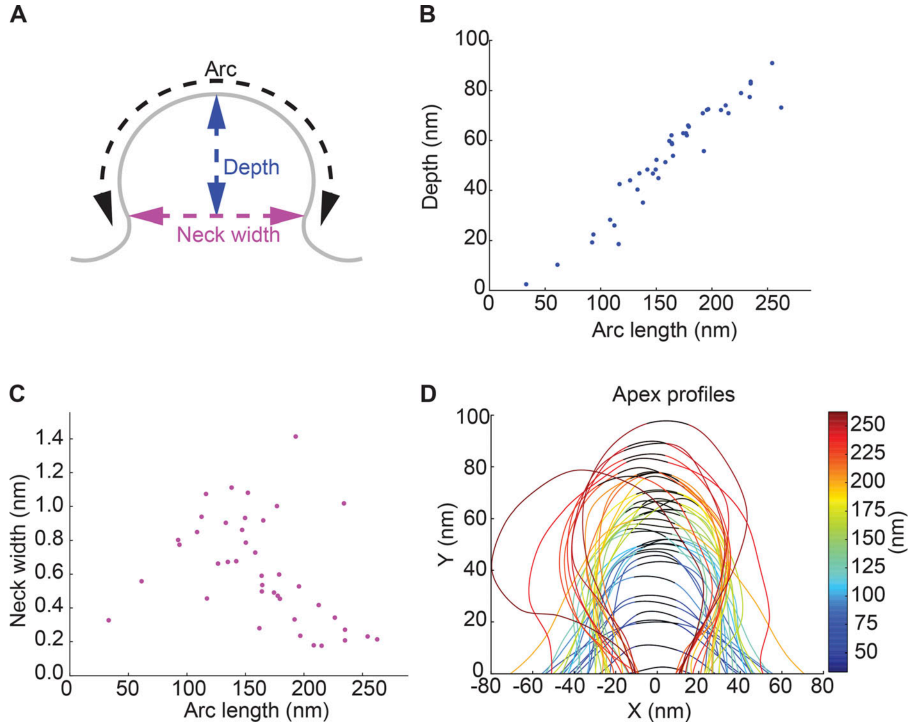


Figure S5. **Changes of CCP depth and neck width during CCP development. (A)** Schematic diagram of the CCP membrane. **(B)** Increase in CCP depth during CCP development. **(C)** Changes in neck width during CCP development. **(D)** CCP apex profiles. The apex is highlighted in black.

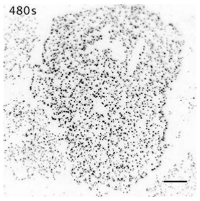


Video 1. **TIRF video of CCP assembly on membrane sheets.** CCP assembly induced by cytosol. Clathrin-AF555 was added to cytosol to visualize CCP. Images were taken at 5 s/frame. Scale bar, 10 µm.

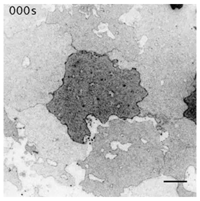


Video 2. **TIRF video of TfR-pHluorin sequestration.** Membrane sheet expressing TfR-pHluorin was incubated with cytosol. Images were taken at 5 s/frame. Scale bar, 10 µm.

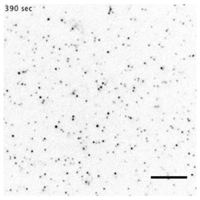


Video 3. **TIRF video of a single molecule of clathrin during CCP assembly.** Clathrin-AF647 (0.5 nM) was added to cytosol to trace single-molecule dynamics. Images were taken at 5 s/frame and played at 30 frames/s. Scale bar, 10 µm.

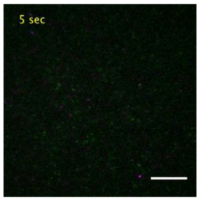


Video 4. **Two-color TIRF video of clathrin turnover during CCP assembly.** Clathrin-AF647 (0.5 nM, magenta) was added to cytosol to trace single-molecule dynamics. Clathrin-AF555 (95 nM, green) was added to cytosol to mark CCP. Images were taken at 5 s/frame and played at 10 frames/s. Scale bar, 10 µm.
